# Sequence-dependent off-target inhibition of TLR7/8 sensing by synthetic microRNA inhibitors

**DOI:** 10.1093/nar/gku1343

**Published:** 2014-12-24

**Authors:** Soroush T. Sarvestani, H. James Stunden, Mark A. Behlke, Samuel C. Forster, Claire E. McCoy, Michelle D. Tate, Jonathan Ferrand, Kim A. Lennox, Eicke Latz, Bryan R.G. Williams, Michael P. Gantier

**Affiliations:** 1 Centre for Cancer Research, MIMR-PHI Institute of Medical Research, Clayton, Victoria 3168, Australia; 2 Department of Molecular and Translational Science, Monash University, Clayton, Victoria 3168, Australia; 3 Institute of Innate Immunity, Biomedical Center, University Hospitals Bonn, Bonn 53127, Germany; 4 Integrated DNA Technologies Inc., Coralville, IA 52241, USA; 5 Host-Microbiota Interactions Laboratory, Wellcome Trust Sanger Institute, Hinxton, CB10 1SA, UK; 6 Centre for Innate Immunity and Infectious Diseases, MIMR-PHI Institute of Medical Research, Clayton, Victoria 3168, Australia; 7 Division of Infectious Diseases & Immunology, University of Massachusetts Medical School, Worcester, MA 01605, USA; 8 Deutsches Zentrum für Neurodegenerative Erkrankungen, Bonn 53127, Germany

## Abstract

Anti-microRNA (miRNA) oligonucleotides (AMOs) with 2′-O-Methyl (2′OMe) residues are commonly used to study miRNA function and can achieve high potency, with low cytotoxicity. Not withstanding this, we demonstrate the sequence-dependent capacity of 2′OMe AMOs to inhibit Toll-like receptor (TLR) 7 and 8 sensing of immunostimulatory RNA, independent of their miRNA-targeting function. Through a screen of 29 AMOs targeting common miRNAs, we found a subset of sequences highly inhibitory to TLR7 sensing in mouse macrophages. Interspecies conservation of this inhibitory activity was confirmed on TLR7/8 activity in human peripheral blood mononuclear cells. Significantly, we identified a core motif governing the inhibitory activity of these AMOs, which is present in more than 50 AMOs targeted to human miRNAs in miRBaseV20. DNA/locked nucleic acids (LNA) AMOs synthesized with a phosphorothioate backbone also inhibited TLR7 sensing in a sequence-dependent manner, demonstrating that the off-target effects of AMOs are not restricted to 2′OMe modification. Taken together, our work establishes the potential for off-target effects of AMOs on TLR7/8 function, which should be taken into account in their therapeutic development and *in vivo* application.

## INTRODUCTION

MicroRNAs (miRNAs) are short single-stranded RNAs (ssRNAs) of ∼21–23 nucleotides that are involved in most regulatory processes necessary for cellular function. These small RNAs fine-tune gene expression through the recruitment of the RNA-induced silencing complex (RISC) to messenger RNA (mRNA) sequences with some degree of complementarity (1). Recruitment of miRNA-RISC to target mRNA sites results in decreased protein synthesis through translational repression and mRNA destabilization (2,3). Because of their ability to bind cognate sequences, the effect of miRNAs on target mRNAs can be directly inhibited through the use of steric antisense oligonucleotides (ASOs) with high affinity for specific miRNAs (4).

ASOs have been used for more than three decades to antagonize mRNA expression and were rapidly adapted to block miRNAs following the rapid expansion of the miRNA field (5,6). Building on knowledge from ASOs, anti-miRNA oligonucleotides (AMOs) bearing 2′-O-Methyl (2′OMe) ribose modification were shown to decrease RISC activity and block miRNA activity (5,6). 2′OMe modification of RNA is common in the field of ASOs for its imparting of nuclease resistance and increased *T*_m_, conferring higher binding affinity for complementary RNA. To date, 2′OMe-modified AMOs remain one of the most widely used tools to dissect miRNA function due to their relatively high affinity for target miRNAs, lack of significant toxicity and low cost of synthesis (7).

In addition to the beneficial use of 2′OMe in ASOs and AMOs, this ribose modification mimics that of endogenous capped mRNAs with the added benefit of decreased immunogenicity (8,9). As such, RNA sensing by innate immune sensors, such as Toll-like receptor 7 (TLR7) is strongly decreased by 2′OMe incorporation in otherwise immunostimulatory oligonucleotides (9–12). In fact, 2′OMe oligonucleotides appear to bind more avidly to TLR7, thereby antagonizing sensing of other immunostimulatory RNAs (13).

TLR7 and TLR8 are specialized in the detection of pathogenic RNA by immune phagocytes, including plasmacytoid dendritic cells and macrophages. Their activation leads to the induction of a potent immune response mediated by pro-inflammatory cytokines, including interferon alpha (IFN-α) and tumour necrosis factor alpha (TNF-α) (14). This innate immune response is essential for mounting an appropriate adaptive immune response to viral infections, such as retroviruses or influenza, and bacterial infections (15–18). Importantly, TLR7 and TLR8 are not activated by all RNAs equally, and uridine-rich sequences are more immunostimulatory than other sequences (19,20). In addition, structural variations in the RNAs can affect TLR7/8 sensing, making it difficult to predict which RNA sequences are immunostimulatory (20,21). While the direct inhibitory effect of as few as one 2′OMe residue incorporated within otherwise immunostimulatory RNAs has been described (9,13,17,22), it is not known whether RNAs that are fully 2′OMe-modified have sequence-dependent effects on the sensing of immunostimulatory pathogenic RNA. Nonetheless, it has previously been reported that incorporation of 2′OMe cytidine residues in RNA did not inhibit TLR7/8 sensing (10,22). This suggests that cytidine-rich 2′OMe RNAs might have less inhibitory activity compared to 2′OMe sequences of similar length with only a few cytidines.

Here, while originally aiming at defining the regulatory roles of the *miR-17∼92* cluster of miRNAs in TLR7 sensing in mouse macrophages, we observed a strong off-target effect from our non-targeting control 2′OMe AMO, indicative of sequence-dependent activity of 2′OMe AMOs on TLR7/8 sensing. We demonstrate that AMOs that are fully 2′OMe-modified inhibit TLR7/8 sensing in a sequence-dependent manner, independent of their miRNA-targeting function. We establish that this effect relies on an inhibitory motif present in more than 50 miRNA AMOs, and that LNA/DNA phosphorothioate AMOs can also inhibit TLR7 in a sequence-dependent manner.

## MATERIALS AND METHODS

### Ethics statement

The use of animals and experimental procedures were approved by Monash Medical Centre Ethics Committee under reference MMCA 2011/25.

### Cell isolation and culture

*miR-17∼92^flox/flox^* mice (Jax mice stock 8458 – on a mixed C57BL/6 and 129S4 background) harbouring *loxP* sites on each side of the miR-17∼92 cluster (*Mir17*,*Mir18*,*Mir19a*,*Mir20a*,*Mir19b-1*,*Mir92–1*) (23), were bred to *LysMCre* mice (kind gift from Dr. Irmgard Förster, LIMES Institute, Bonn, Germany) expressing the Cre protein from the myeloid-specific lysozyme 2 gene (*Lyz2*) promoter (24). Primary bone marrow extraction and differentiation to generate bone marrow-derived macrophages (BMMs) were carried out following standard procedures. Briefly, femurs from wild-type (WT) C57BL/6 and *miR-17∼92^flox/flox^* ×*LysMCre* mice were flushed with Dulbecco's modified Eagle's medium (DMEM) complemented with 1× antibiotic/antimycotic and 10% fetal bovine serum (FBS) (complete DMEM), and cells were plated in complete DMEM supplemented with 20% L-929 cell-conditioned medium in T75-treated flasks for 6 days at 37°C in a 5% CO_2_ atmosphere (and rinsed with fresh medium on day 3). On day 6, the cells were collected and plated at 80 000 cells per well of a 96-well plate in 150 μl DMEM complete with L-929 cell-conditioned medium, and incubated overnight prior to stimulation on day 7. Human blood was collected from healthy donors in heparin-treated tubes. Peripheral blood mononuclear cells (PBMCs) were purified with Ficoll-Paque plus (GE Healthcare) as previously reported (25). PBMCs were plated at 200 000 per well of a 96-well plate in 150 μl of RPMI 1640 plus L-glutamine medium (Life Technologies), complemented with 1× antibiotic/antimycotic and 10% FBS (Life Technologies), referred to as complete RPMI. The cells were incubated for 2–4 h at 37°C in 5% CO_2_ atmosphere before transfection with AMOs and stimulation by TLR agonists. HEK293 TLR7 and TLR9 cells stably expressing human TLR7 or 9 were purchased from Invivogen (293XL/hTLR7-HA and 293XL/hTLR9-HA) and maintained in complete DMEM supplemented with 10 μg/ml blasticidin.

### Synthetic RNAs/AMOs and TLR ligands for stimulation of cells

All synthetic RNAs were synthesized by Integrated DNA Technologies (IDT). Immunostimulatory B-406AS-1 (UAAUUGGCGUCUGGCCUUCUU) (20) and β-Gal-656-REV (AAAUCGCUGAUUUGUGUAGUU) (12), and non-stimulatory control RD (UAACACGCGACAGGCCAACUU) ssRNAs with no backbone modification were resuspended in duplex buffer (100 mM potassium acetate, 30 mM HEPES, pH 7.5, DNase–RNase-free H_2_O) to 80 μM. AMOs complementary to human miRNAs were resuspended in sterile RNase-free TE buffer (Life Technologies) to 4 μM. 2′OMe AMOs were synthesized with 2′OMe groups (‘M’), phosphodiester linkages (unless otherwise noted) and a non-nucleotide napthyl-azo group chemical modifier (dubbed ‘ZEN’- ‘z’) near both ends (MzMMMMMMMMMMMMMMMz), increasing potency at low nanomolar concentrations (7) (Supplementary Table S1). LNA/DNA AMOs were synthesized with phosphorothioate linkages, as detailed in Supplementary Table S1. AMOs and ssRNAs were transfected with DOTAP (Roche) and pure RPMI in biological triplicate, as previously described (20–21,25). The ratios of DOTAP to RNA (at 80 μM) were as follows: 3.5 μg/μl of ssRNA in BMMs and 5 μg/μl of ssRNA in PBMCs. DOTAP-complexed AMOs were added 30–45 min before the addition of other TLR ligands or DOTAP-complexed immunostimulatory ssRNA. Following stimulation, the samples were incubated overnight for 16–18 h before cytokine measurement by enzyme-linked immunosorbent assay (ELISA). R848 (chemical TLR7/8 agonist), naked polyI:C (TLR3 agonist) and ODN 2006 (human TLR9 agonist) (Invivogen) were directly added to medium to a final concentration of 1–2 μg/ml (R848), 20 μg/ml (polyI:C) or 400 nM (ODN 2006), or as otherwise noted. ‘Mock’ condition refers to DOTAP only treatment. Stimulations were carried out in biological triplicate in all experiments.

### Detection of cytokines

Human TNF-α and mouse TNF-α were measured using BD OptEIA ELISA sets, according to the manufacturer's instructions (BD Biosciences). Human IFN-α detection was carried out as previously reported (26). Tetramethylbenzidine substrate (Sigma Aldrich) was used for quantification of the cytokines on a Fluostar OPTIMA (BMG LABTECH) plate-reader.

### Low-density miRNA arrays

TaqMan^®^ Array Rodent MicroRNAs (A Cards v2.0, Life Technologies) were used for the estimation of miRNA abundance in BMMs. Total RNA containing small RNAs was purified from BMMs using the mirVana miRNA isolation kit (Life Technologies). Briefly, 300 ng of total RNA containing small RNAs was reverse transcribed using the Megaplex™ RT Primers, Rodent Pool A (Life Technologies) with the TaqMan^®^ MicroRNA Reverse Transcription Kit and each plate was consecutively run using the TaqMan^®^ Universal Master Mix II on the 7900 RT-PCR system, according to the manufacturer's instructions. Relative miRNA abundance determination relied on the assumption that each miRNA TaqMan^®^ assay detected miRNAs with assimilable amplification efficiencies. The *C*_qs_ (*C*_q_ = quantification cycle) obtained for each miRNA were used to directly infer the abundance of each miRNA relative to that of the most abundant miRNA (miR-222). This was achieved by subtracting individual *C*_q_ values from that obtained for miR-222 (e.g. Δ*C*_q_ = *C*_q_ miR-21 – *C*_q_ miR-222). The relative level of each miRNA to miR-222 was then inferred using 2^(-Δ*C*q)^.

### miRNA reverse transcription quantitative real-time polymerase chain reaction (RT-qPCR)

Total RNA containing small RNAs was purified from cultured cells using the miRNeasy kit (Qiagen). miRNA TaqMan^®^ assays (Applied Biosystems) for the indicated miRNAs were used according to the manufacturer's instructions, where 10–30 ng of total RNA was reverse transcribed with pools of miRNA-specific reverse transcription primers. miRNA levels were determined with the TaqMan^®^ Universal PCR Master Mix on a 7900 RT-PCR system (Life Technologies), and fold changes in expression were calculated by the 2^−ΔΔ*C*q^ method using U6 snRNA (#001973) as reference gene.

### Luciferase assays

HEK293 cells stably expressing TLR7 or 9 were transfected with pNF-κB-Luc4 reporter (Clontech) with lipofectamine 2000 (Life Technologies), 24–48 h before transfection with AMOs and TLR ligand stimulation. Following overnight stimulation with TLR ligand, the cells were lysed in 40 μl (for a 96-well plate) of passive lysis buffer 1× for 20 min at room temperature. Note that15 μl of the lysate was then subjected to firefly luciferase assay using 40 μl of Luciferase Assay Reagent (Promega). Luminescence was quantified with a Fluostar OPTIMA (BMG LABTECH) luminometer.

### Statistical analyses

Statistical analyses were carried out using Prism 6 (GraphPad Software Inc.). Every experiment was carried out in biological triplicate, and repeated a minimum of two independent times (giving a minimum of six biological values). One-way and two-way analyses of variance (ANOVA) were used when comparing groups of conditions, while unpaired one-tailed *t*-tests were used when comparing two conditions. Symbols used: *P ≤ 0.05, **P ≤ 0.01, ***P ≤ 0.001, ****P ≤ 0.0001 and ‘ns’ is non-significant.

## RESULTS

### miRNA-independent inhibition of immunostimulatory RNA sensing by 2′OMe AMO

**Figure 1.**
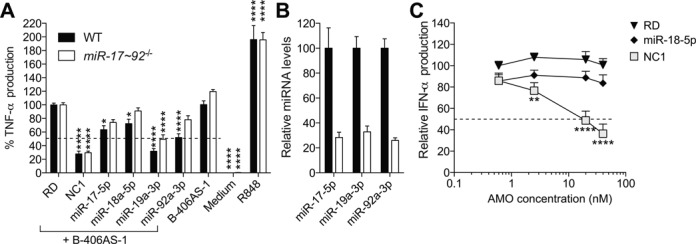
Off-target effect of 2′OMe AMOs on immunostimulatory RNA sensing. (**A**) BMMs from WT mice or *miR-17∼92^flox/flox^* × *LysMCre* mice pre-treated for 45 min with 40 nM of the indicated 2′OMe AMO or non-2′OMe control RNA (RD) were stimulated overnight with 180 nM of immunostimulatory ssRNA (B-406AS-1), and TNF-α levels were measured in supernatants by ELISA. Percentages of TNF-α production compared to the RD+B-406AS-1 condition are given. The data are averaged from three independent experiments, where each treatment was conducted in biological triplicate. R848 was used as a positive control for induction of TNF-α. (**B**) Total RNA was extracted from non-stimulated BMMs, and miR-17-5p, miR-19a-3p and miR-92a-3p levels were measured by RT-qPCR. Expression was normalized to U6 RNA and is shown relative to WT mice. Data are averaged from three independent experiments in biological duplicate. (**C**) Human PBMCs were pre-treated for 45 min with the indicated dose of AMO or control RNA (RD), and stimulated overnight with 180 nM of B-406AS-1, and IFN-α levels were measured in supernatants by ELISA. The data are averaged from three independent experiments in different blood donors, where each treatment was conducted in biological triplicate. Percentages of cytokine production compared to the 0.6 nM RD+B-406AS-1 condition are given. (**A** and **C**) Ordinary two-way ANOVA with Dunnett's multiple comparison tests to the RD+B-406AS-1 condition are shown. Unless otherwise indicated, differences were not significant. (**A**–**C**) SEM is shown.

We have recently reported a positive regulatory role for miR-19 miRNAs (including both miR-19a-3p and miR-19b-3p) in the control of nuclear factor kappa B (NF-κB) signalling in several cell lines (27). To investigate the specific impact of miR-19 inhibition, relative to that of other members of the same cluster of miRNAs (miR-17-5p, miR-18a-5p and miR-92a-3p), we measured the inhibition of TLR7 signalling in primary mouse BMMs treated with specific 2′OMe AMOs. In agreement with our previous results, miR-19a-3p inhibition resulted in ∼70% decreased TNF-α production in response to transfected immunostimulatory ssRNA (B-406AS-1), when compared to the non-2′OMe control RNA sequence (RD) (Figure 1A). miR-92a-3p inhibition also reduced TNF-α production by ∼50%, suggesting its involvement in regulating TLR7-mediated immune induction, while miR-18a-5p and miR-17-5p inhibition only had a modest effect on RNA sensing (Figure 1A). Surprisingly, however, transfection of the non-targeting control (NC1) 2′OMe AMO resulted in the most potent inhibition of TNF-α production, indicative of an off-target effect of this molecule on immunostimulatory ssRNA sensing. In light of previous reports that 2′OMe RNAs can act as TLR7 antagonists (10,11), we reasoned that certain 2′OMe AMO sequences could be more potent than others—thereby explaining the divergent activity of miR-18a-5p and NC1 AMOs. To distinguish the direct contribution of miR-19 and -92 in TLR7 sensing from a potential off-target effect of the 2′OMe AMOs used, experiments were replicated in BMMs from *miR-17∼92^flox/flox^* ×*LysMCre* mice—where levels of mature miR-17-5p, miR-19a-3p and miR-92a-3p were decreased by ∼70% (Figure 1B). Critically, pre-treatment with the miR-19a-3p 2′OMe AMO still reduced TNF-α production by ∼50%, suggesting an activity independent of its miRNA-targeting function (Figure 1A). In accord with the concept of a sequence-dependent and miRNA-independent activity of select 2′OMe AMOs on ssRNA sensing, transfection of 2′OMe NC1 AMO resulted in a dose-dependent inhibition of IFN-α production (indicative of TLR7 recruitment (21,28)) to immunostimulatory ssRNA in human PBMCs, while the miR-18a-5p 2′OMe AMO did not (Figure 1C). Collectively, these results support the sequence-dependent inhibition of immunostimulatory RNA sensing by 2′OMe AMOs, independent of their miRNA-targeting activity.

### Sequence-dependent inhibition of immunostimulatory sensing by 2′OMe AMOs

**Figure 2.**
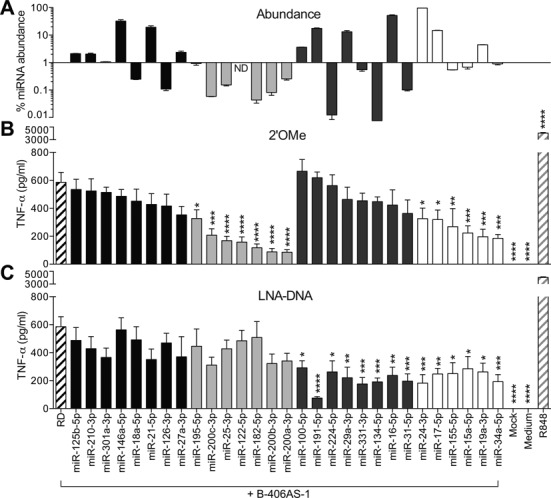
Inhibition of immunostimulatory RNA sensing by 2′OMe AMOs is sequence dependent. (**A**) Total RNA from BMMs on day 8 post-isolation was analysed by TaqMan^®^ Array Rodent MicroRNAs in biological duplicate, and miRNA abundance relative to that of the most abundant miRNA—miR-222—calculated as detailed in the Materials and Methods section. ND, not detected. BMMs from WT mice pre-treated with 40 nM of the indicated 2′OMe AMO (**B**) or LNA/DNA AMO (**C**) for 45 min were stimulated overnight with 180 nM of immunostimulatory ssRNA (B-406AS-1), and TNF-α levels were measured in supernatants by ELISA. The RD+B-406AS-1 condition was used as control. The data are averaged from two independent experiments in biological triplicate. (**B** and **C**) Ordinary one-way ANOVA with Dunnett's multiple comparison tests to the RD+B-406AS-1 condition are shown. Unless otherwise indicated, differences were not significant. Class 1: AMOs with no inhibitory effect (black bars). Class 2: AMOs with inhibitory effect with 2′OMe chemistry only (light grey bars). Class 3: AMOs with inhibitory effect with LNA/DNA chemistry only (dark grey bars). Class 4: AMOs with inhibitory effect with both chemistries (white bars). (**A–C**) SEM is shown.

To define further the modalities of sequence-dependent TLR7 inhibitory activity of 2′OMe AMOs, a panel of 29 AMOs was screened for their potential effect on TLR7 activation by immunostimulatory ssRNA, in mouse primary BMMs. To exclude potential miRNA-specific effects, we performed parallel analyses of these 29 AMOs synthesized with both 2′OMe and LNA/DNA chemistries, and profiled relative miRNA abundance by low-density TaqMan^®^ miRNA microarrays (Figure 2). The results from these experiments could be grouped into four classes of AMOs: (i) AMOs that had no impact on immunostimulatory ssRNA sensing, with either chemistry; (ii) AMOs that only had an inhibitory effect with the 2′OMe chemistry; (iii) AMOs that only had an inhibitory effect with the LNA/DNA chemistry; and (iv) AMOs that had an inhibitory effect with both chemistries. Although the inhibitory effect of select LNA/DNA AMOs was surprising, it is consistent with a previous report that phosphorothioate DNA oligonucleotides can inhibit TLR7 sensing in a sequence-dependent manner (29). The sequence-specific and miRNA-independent significant inhibition of immunostimulatory ssRNA sensing by 2′OMe AMOs targeting miR-195-5p, miR-25-3p, miR-122-5p, miR-200a/b/c-3p and miR182-5p (Figure 2B) was supported by the lack of inhibitory activity with LNA/DNA AMOs (Figure 2C), and the low abundance of these miRNAs (less than 100-fold the level of the most abundant miRNA in BMMs) (Figure 2A).

### Sequence-dependent inhibition of human TLR7/8 sensing by AMOs

**Figure 3.**
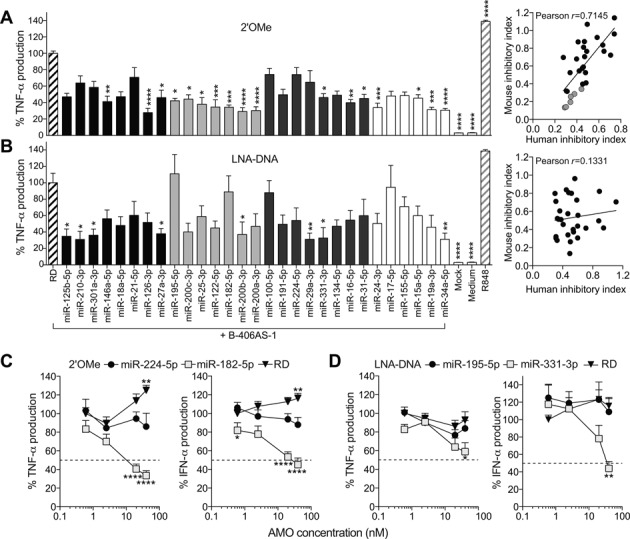
Sequence-dependent inhibition of RNA sensing by 2′OMe AMOs is conserved in human PBMCs. Human PBMCs pre-treated with 40 nM of the indicated 2′OMe AMO (**A**) or LNA/DNA AMO (**B**) for 45 min were stimulated overnight with 180 nM of B-406AS-1. TNF-α levels were measured in supernatants by ELISA. Correlation of relative TNF-α production to RD control condition (i.e. inhibitory index) in both human PBMCs and mouse BMMs is shown for each AMO chemistry (right panels). 2′OMe AMOs of ‘Class 2’ are shown in grey. Correlation for 2′OMe AMOs was highly significant (*P* < 0.0001). (**C** and **D**) Human PBMCs were pre-treated for 45 min with the indicated dose of AMO or RD, and stimulated overnight with 180 nM of B-406AS-1. (**C** and **D**) TNF-α and IFN-α levels were measured in supernatants by ELISA. (**A–D**) The data are averaged from a minimum of three independent experiments in different blood donors, in biological triplicate. Percentages of cytokine production compared to the RD+B-406AS-1 condition (**A** and **B**) or the 0.6 nM RD+B-406AS-1 condition (**C** and **D**) are given. Ordinary one-way (**A** and **B**) or two-way (**C** and **D**) ANOVA with Dunnett's multiple comparison tests to the RD+B-406AS-1 (**A** and **B**), the miR-224-5p+B-406AS-1 (**C**) or the miR-195-5p+B-406AS-1 (**D**) conditions are shown. (**A**–**D**) Unless otherwise indicated, differences were not significant. SEM is shown.

To extend these results, we investigated the inhibitory activity of the panel of 2′OMe and LNA/DNA AMOs on TLR7/8 sensing in human PBMCs. The *in vitro* system using human PBMCs allows for the analysis of TLR8 activation, as this function in RNA sensing is not conserved in mouse BMMs (14). In this model, assessment of TNF-α production in response to immunostimulatory ssRNAs reflects TLR8 activation, while IFN-α production reflects TLR7 activation (21,28). In agreement with a TLR8 inhibitory activity of select 2′OMe AMOs, TNF-α production was significantly decreased with all seven 2′OMe AMOs from Class 2 (Figure 3A). Noteworthy, select AMOs from Classes 1 and 3 (which did not significantly affect TNF-α production in BMMs) significantly reduced TNF-α production, suggesting a specific TLR8 inhibitory activity of these sequences (e.g. 2′OMe AMOs for miR-146a-5p or miR-331-3p). Nonetheless, there was a significant correlation between the inhibitory activities of the 2′OMe AMOs on TNF-α production in human PBMCs and mouse BMMs (*r* = 0.7145), indicating conservation of the inhibitory activity of the 2′OMe AMOs between TLR7 and TLR8 (Figure 3A). For LNA/DNA AMOs, the profile of TNF-α inhibition differed notably from that seen in BMMs, with only two AMOs from Class 3 significantly reducing cytokine production (Figure 3B). Consequently, there was no significant correlation between TNF-α inhibition in human PBMCs and mouse BMMs for LNA/DNA AMOs, suggesting a different activity on TLR7 and TLR8 (Figure 3B). Next, dose-response studies comparing the activity of highly inhibitory AMOs (miR-182-5p 2′OMe and miR-331-3p LNA/DNA) and that of poorly inhibitory AMOs (miR-224-5p 2′OMe and miR-195-5p LNA/DNA) on both TLR7 and TLR8 sensing were conducted in human PBMCs (Figure 3C and D). The miR-182-5p 2′OMe AMO had a strong inhibitory effect on both TNF-α and IFN-α, and was significantly more inhibitory than the miR-224-5p 2′OMe AMO in a dose-dependent manner (Figure 3C). Similar results were seen with miR-195-5p 2′OMe AMO dose-response studies (Supplementary Figure S1). In line with a predominant TLR7 inhibitory effect of LNA/DNA AMOs, the miR-331-5p LNA/DNA AMO significantly decreased IFN-α levels compared to the miR-195-5p LNA/DNA AMO, while having a smaller impact on TLR8-driven TNF-α production (Figure 3D). Given a previous report that the inhibitory activity of LNA/DNA AMOs could be related to the phosphorothioate (PS) backbone of these molecules (30), we studied a variant of miR-224-5p 2′OMe synthesized with a PS backbone (miR-224-5p 2′OMe PS) in human PBMCs. miR-224-5p 2′OMe AMO was selected for its low inhibitory activity, while its LNA/DNA variant significantly inhibited TLR7 in mouse BMMs (Figure 2). miR-224-5p 2′OMe PS increased inhibition of TNF-α production compared to the parent phosphodiester molecule (miR-224-5p 2′OMe), similar to that of the miR-224-5p LNA/DNA variant (also synthesized with a PS backbone), in human PBMCs (Supplementary Figure S2A). However, there was no significant impact of the PS backbone on IFN-α production, indicating a TLR8-specific inhibitory activity of the PS backbone in the context of this sequence (Supplementary Figure S2A). Collectively, these results suggest that while 2′OMe AMOs can inhibit both TLR7 and TLR8, LNA/DNA AMOs do not equally affect these two receptors—with a preferential effect on TLR7 inhibition, and contribution of the PS backbone to TLR8 inhibition.

### Inhibition of immunostimulatory RNA sensing by 2′OMe AMOs is governed by a specific motif

**Figure 4.**
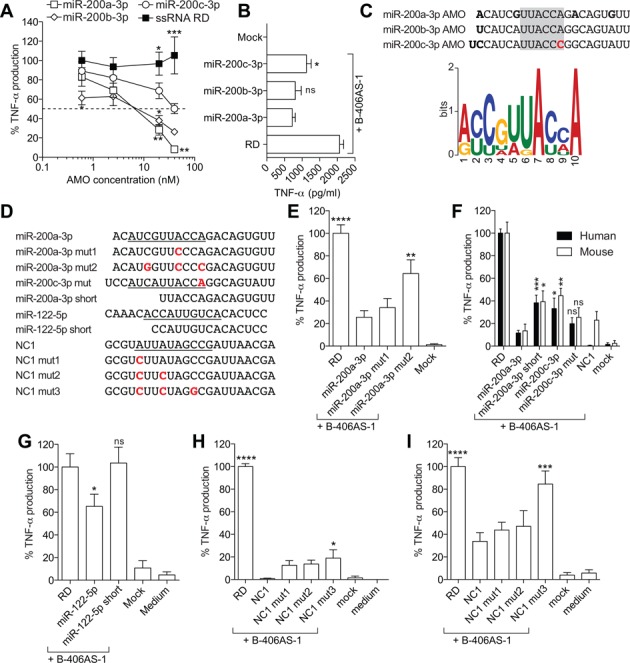
Inhibition of RNA sensing by 2′OMe is motif dependent. (**A**) BMMs from WT mice pre-treated with the indicated dose of AMO or RD for 45 min were stimulated overnight with 180 nM of B-406AS-1, and TNF-α levels were measured in supernatants by ELISA. The data are averaged from three independent experiments in biological triplicate. Percentages of cytokine production compared to the 0.6 nM RD+B-406AS-1 condition are given. Ordinary two-way ANOVA with Dunnett's multiple comparison tests to miR-200c-3p+B-406AS-1 are shown. (**B**) Human PBMCs were pre-treated for 45 min with the indicated dose of AMO or RD, and stimulated overnight with 180 nM of B-406AS-1. TNF-α levels were measured in supernatants by ELISA. The data are averaged from three independent experiments in different blood donors, in biological triplicate. Unpaired *t*-tests comparing miR-200a-3p+B-406AS-1 and indicated conditions are shown. (**C**) Top: alignment of miR-200a/b/c-3p AMOs with the core inhibitory motif highlighted in grey. Bottom: MEME pictogram of the relative frequency of bases constituting the inhibitory motif (40). (**D**) Alignment of miR-200a-3p, miR-200c-3p, miR-122-5p and NC1 AMO variants with altered/truncated inhibitory motifs (in 5′-3′ orientation). Native inhibitory motifs are underlined and point mutations are in red bold. BMMs from WT mice (**E, F, G, I**) or human PBMCs (**F, H**) pre-treated with 40 nM of the indicated 2′OMe AMO or RD were stimulated overnight with 180 nM of B-406AS-1, and TNF-α levels were measured in supernatants by ELISA. Percentages of cytokine production compared to the RD+B-406AS-1 condition are given. The data are averaged from a minimum of two independent experiments, in biological triplicate. Ordinary one-way ANOVA with Dunnett's multiple comparison tests to miR-200a-3p+B-406AS-1 (**E**) or NC1+B-406AS-1 (**H** and **I**) are shown. Unpaired *t*-tests comparing miR-200a-3p+B-406AS-1 (**F**) or RD+B-406AS-1 (**G**) and indicated conditions are shown (ns, not significant). (**A, E, H, I**) Unless otherwise indicated, differences were not significant. (**A, B** and **E**–**I**) SEM is shown.

We noted that miR-200a-3p, miR-200b-3p and miR-200c-3p 2′OMe AMOs had a strong inhibitory effect on immunostimulatory ssRNA sensing in primary BMMs. Given their low abundance (Figure 2A), this is likely independent of miRNA-targeting activities. Importantly, although miR-200a-3p and miR-200b-3p had similar inhibitory activities, miR-200c-3p was a less potent inhibitor (Figure 2B). These results were confirmed in dose-response studies in primary BMMs, demonstrating that the miR-200a-3p 2′OMe AMO was significantly more inhibitory than the miR-200c-3p AMO (Figure 4A). Similar results were found with immunostimulatory ssRNA sensing in human PBMCs on TLR8, where the miR-200c-3p AMO was also significantly less potent than the miR-200a-3p AMO at reducing TNF-α production (Figure 4B). Notably, as suggested by their name, these miRNAs belong to the same family of miRNAs, with strong sequence conservation. Given that the miR-200b-3p and miR-200c-3p AMOs only differ by two bases, we hypothesized that one of these two variations in the miR-200c-3p AMO was responsible for the decreased TLR7-inhibitory activity (Figure 4C). Relying on the variations between the miR-200a-3p and miR-200b-3p AMOs, we defined a central core UUACCA sequence changed to UUACCC in miR-200c-3p, which is potentially directly involved in the inhibitory activity of these 2′OMe AMOs on TLR7 sensing (Figure 4C). Critically, this core sequence overlapped with a significantly enriched motif found in all the inhibitory sequences of Class 2 AMOs previously identified, in 5′-3′ orientation (for miR-200a/b-3p, and miR-25-3p) or 3′-5′ orientation (for AMO-NC1, miR-182-5p, miR-122-5p and miR-195-5p) (Figure 4C and Supplementary Table S2). To validate the TLR7-inhibitory function of this motif in the miR-200 2′OMe AMOs, we synthesized two AMO variants of the miR-200a-3p 2′OMe AMO, with one (miR-200a-3p mut1) and three (miR-200a-3p mut2) base changes in the most conserved residues of the enriched motif (Figure 4C and D). While a single base change in miR-200a-3p mut1 did not significantly affect the inhibitory activity of this 2′OMe AMO, the addition of another two changes significantly compromised the inhibitory effect of the miR-200a-3p mut2 2′OMe AMO in primary BMMs (Figure 4E), thereby underlining a critical role of this motif. To define further the impact of this motif in the modulation of TLR7/8 sensing, we generated a set of AMO mutants and truncated variants (Figure 4D) based on miR-200a/c-3p, miR-122-5p and NC1 2′OMe AMOs. While the native miR-200c-3p 2′OMe AMO was significantly less inhibitory than miR-200a-3p 2′OMe AMO, activity of its mutated variant miR-200c-3p mut (restoring the core UUACCA sequence) did not significantly differ from that of miR-200a-3p in either human PBMCs or mouse BMMs (Figure 4F). In addition, truncation of miR-200a-3p 2′OMe AMO to a shorter form (miR-200a-3p short) directly impacting on the 5′ end of the inhibitory motif, also resulted in decreased inhibitory activity of miR-200a-3p in both human and mouse (Figure 4F). Similar results were found for a 15mer variant of miR-122–5p (Figure 4G). In addition, multiple variations of the predicted inhibitory motif in NC1 mut3 significantly impacted on the inhibitory activity of 2′OMe AMO NC1, in both human and mouse (Figure 4H and I). Nonetheless, NC1 mut3 retained a stronger inhibitory activity in human PBMCs than in mouse BMMs, suggesting that human TLR8 inhibition was less dependent on this motif than mouse TLR7 (Figure 4H and I). Altogether, these findings establish the importance of the motif identified in the inhibitory activity of 2′OMe AMOs on immunostimulatory RNA sensing.

### Motif-dependent 2′OMe AMO inhibitory activity is TLR7 specific

**Figure 5.**
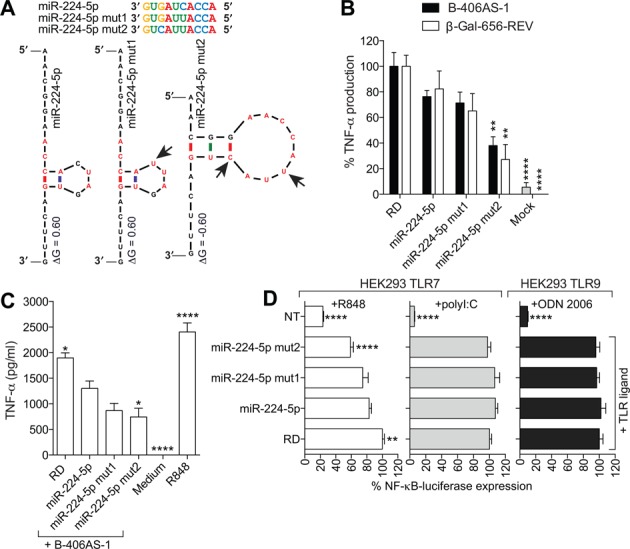
Motif-dependent activity of 2′OMe AMO is TLR7 specific. (**A**) mFOLD-predicted structure and free energy (Δ*G*, in kcal/mol) of miR-224–5p 2′OMe AMO used at 37°C (31). The bases of the consensus inhibitory motif are shown in red. Arrows point to mutations introduced in miR-224–5p AMOs to create the inhibitory motif in miR-224–5p mut2. BMMs from WT mice (**B**) and human PBMCs (**C**) pre-treated with 40 nM of the indicated 2′OMe AMO or RD, were stimulated overnight with 180 nM of B-406AS-1 (**B** and **C**) or β-Gal-656-REV (**B**), and TNF-α levels were measured in supernatants by ELISA. (**B**) The data are averaged from two (for β-Gal-656-REV) or three (for B-406AS-1) independent experiments, in biological triplicate. Percentages of cytokine production compared to the RD+B-406AS-1 (black) or β-Gal-656-REV condition (white) are given. (**C**) The data are averaged from three independent experiments in different blood donors, in biological triplicate. (**D**) HEK293 cells stably expressing TLR7 or TLR9 and an NF-κB-luciferase reporter, were transfected with 200 nM of the indicated AMO for 45 min prior to stimulation with R848 (200 ng/ml) (white) or polyI:C (20 μg/ml) (grey) for TLR7 cells, and ODN 2006 (400 nM) (black) for TLR9 cells. Firefly luciferase was assayed after overnight incubation as described in the Materials and Methods section. Luminescence reads normalized to unstimulated cells (NT) are shown as percentages of the RD+TLR ligand condition. The data are averaged from a minimum of three independent experiments in biological triplicate. (**B**–**D**) Ordinary one-way ANOVA with Dunnett's multiple comparison tests to the miR-224–5p+B-406AS-1 condition are shown. Unless otherwise indicated, differences were not significant. SEM is shown.

The above results led us to assess whether base changes to the poorly inhibitory miR-224-5p 2′OMe AMO, introducing the consensus inhibitory motif, could increase its inhibitory activity on TLR7. Two variants of the miR-224-5p 2′OMe AMO were synthesized, with one or two base changes restoring the most highly conserved residues of the motif otherwise lacking in miR-224-5p (Figure 5A). Notably, the miR-224-5p mut2 2′OMe AMO showed an increase in the mFOLD-predicted secondary structure stability when compared to the native sequence and the other mutant (mut1) (31). In line with an essential role for the motif in the inhibitory activity of 2′OMe AMOs, the miR-224-5p mut2 2′OMe AMO was significantly more inhibitory than the native sequence on the sensing of two different immunostimulatory ssRNAs (B-406AS-1 and β-Gal-656-REV) in mouse primary BMMs (Figure 5B). The inhibitory effect of miR-224-5p mut2 was also seen on TLR8 in human PBMCs, with reduced TNF-α levels compared to the native 2′OMe AMO (Figure 5C). To confirm the specific activity of the miR-224-5p mut2 2′OMe AMO variant on TLR7, the impact of these AMOs was studied in HEK293 cells stably expressing TLR7 or TLR9, and a reporter of downstream NF-κB activity. Noteworthy, HEK293 cells produce endogenous levels of TLR3, and TLR7-expressing cells can also be stimulated by polyI:C to trigger TLR3-dependent NF-κB activation. Transfection of the miR-224-5p mut2 but not the mut1 2′OMe AMO resulted in a significantly increased inhibition of NF-κB activation by the chemical TLR7/8 agonist R848 in HEK293 TLR7 cells, but not with polyI:C treatment, compared to the native miR-224-5p 2′OMe AMO (Figure 5D). In addition, transfection of the miR-224-5p mut2 2′OMe AMO had no impact on TLR9 stimulation by ODN 2006. Collectively, these results establish the sequence-dependent effect of 2′OMe AMOs on TLR7/8 inhibition, independent of their miRNA-targeting function.

## DISCUSSION

Given their widespread regulatory role in most aspects of cellular biology, miRNAs present immense therapeutic potential. Since miRNAs can regulate gene networks rather than specific targets, they have strong biological phenotypes through their compounded effects on many genes (32). For example, we recently identified a network of miR-19 targets converging on the control of inflammation by virtue of regulating the expression of components of the NF-κB signalling pathway (27). While transfection of exogenous synthetic miRNAs can present the same constraints as small interfering RNAs regarding off-target effects, the *in vivo* application of the two technologies is principally hindered by delivery and stability issues (33). Conversely, strategies aimed at blocking miRNAs arguably present greater therapeutic potential in the immediate future, as they are based on highly modified nucleic acids, building on ASO technology. This is illustrated with the example of the anti-miR-122-5p miRNA, miravirsen, a 15-nucleotide LNA/DNA phosphorothioate AMO with demonstrated therapeutic effect against hepatitis C virus in recent human clinical trials (34).

High-affinity ASOs with certain types of modifications, such as LNA bases, are already known to have hepatotoxic potential, which is directly dependent on their sequence and modification pattern (35). It is therefore anticipated that LNA-containing AMOs will have sequence-dependent hepatotoxic potential. In contrast, 2′OMe-modified AMOs do not exhibit the toxicity associated with LNA/DNA phosphorothioate AMOs, and can be modified at their ends to block exonuclease activities and increase stability in serum (7,36–37). Such modifications can include the use of double-stranded RNA flanking regions (36) or chemical modifications, such as phosphorothioate or the recently reported N,N-diethyl-4-(4-nitronaphthalen-1-ylazo)-phenylamine (ZEN) (7,37). With the observation that 2′OMe modification could be used to ablate TLR7/8 activation by synthetic RNAs (11,38), 2′OMe incorporation in synthetic RNAs has the significant added advantage of abrogating the potential off-target activation of innate immunity by RNA AMOs, which would otherwise induce TLR7/8 in a sequence-dependent manner. Importantly, although two reports have previously indicated that the inhibitory activity of 2′OMe modifications could be sequence dependent (with 2′OMe cytidine residues having no significant inhibitory activity) (10,22), there are no accounts regarding the activity of different sequences of fully 2′OMe-modified RNA on other immunostimulatory RNAs.

In this work, we originally set out to study the role of the individual members of the miR-17∼92 cluster of miRNAs (miR-17/20a, miR-19a/b, miR-18a and miR-92a) on TLR7-driven NF-κB signalling in mouse primary macrophages. In accord with our previous findings (27), we demonstrated that 2′OMe AMO-mediated blocking of miR-19 significantly reduced the production of TNF-α induced by immunostimulatory ssRNA in WT BMMs. However, this effect of the miR-19a-3p 2′OMe was mostly retained in BMMs depleted of miR-19a-3p and miR-19b-3p, indicative of a miRNA-independent effect (Figure 1). In addition, we observed a strong off-target effect of the non-targeting control 2′OMe AMO on TNF-α production (Figure 1). Taken together with our later observations that targeting of the liver-specific miR-122-5p or poorly abundant miR-195-5p, miR-25-3p, miR-200a/b/c-3p, miR182-5p and the mutant miR-224-5p mut2 by 2′OMe AMOs (but not their LNA/DNA AMO counterparts) also resulted in significant inhibition of immunostimulatory ssRNA sensing, our work establishes sequence-dependent and miRNA-independent off-target inhibitory activity of 2′OMe AMOs on the immune sensing of pathogenic RNA by human and mouse phagocytes. The inhibitory effect was dose dependent, with a maximum IC_50_ around 2 nM for miR-195-5p 2′OMe AMO (Supplementary Figure S1), while other 2′OMe AMOs (for example, miR-224-5p, not shown) showed only minor inhibitory activity when used at concentrations as high as 80 nM. Given the molarity of immunostimulatory ssRNA used in our experiments (at 180 nM), the results suggest that these specific sequences are active at 1/90th of stimulatory ssRNA concentration, in line with the findings of others (13).

DOTAP-mediated transfection of immunostimulatory RNA sequences in mouse BMMs instigates a TLR7-dependent TNF-α production, as evidenced by a lack of response in cells from *TLR7*-deficient mice (20–21,39). To directly implicate TLR7 in sequence-dependent activity of 2′OMe AMOs, we further demonstrated that NF-κB activation was impaired by an inhibitory variant of miR-224-5p 2′OMe AMO, in HEK293 TLR7 cells stimulated with the TLR7/8 chemical agonist R848. This effect of selected 2′OMe AMOs was specific to TLR7 and was not seen when the HEK293 TLR7 cells were stimulated with the TLR3 ligand polyI:C, or in HEK293 TLR9 cells stimulated with a TLR9 agonist. Importantly, we also observed inhibitory activity with the native miR-224-5p 2′OMe in HEK293 TLR7 cells, with doses ≥200 nM. We anticipate that high doses of most 2′OMe AMOs will antagonize TLR7 sensing, independent of the sequence used. However, even at 200–400 nM, miR-224-5p mut2 had a stronger inhibitory effect on TLR7 than the native miR-224-5p AMO (Figure 5 and not shown), underlining detectable sequence-dependent effects between AMOs used at high doses.

While comparing the effects of miR-200 AMO variants, we noted that miR-200c-3p had a lesser capacity to inhibit TNF-α production, compared to miR-200a-3p and miR-200b-3p. Given the similarity of the sequences, we were able to identify a central region (UUACCA) that was not conserved in miR-200c-3p and could be at play in the inhibitory activity of these molecules. Critically, we found this motif in miR-182-5p and miR-122-5p AMOs, when read in 3′-5′ orientation. Relying on Multiple Em for Motif Elicitation (MEME) analysis of the inhibitory 2′OMe AMO sequences of Class 2 in either 5′-3′ or 3′-5′ orientation (40), we demonstrated that they all contained a longer motif derived from this UUACCA (Figure 4 and Supplementary Table S2). Mutation or deletion of part of this motif resulted in the loss of inhibitory activity for miR-200a-3p, miR-122-5p and NC1 2′OMe AMO variants, while introduction of this motif to the poorly inhibitory miR-224-5p AMO significantly increased the inhibitory activity of the AMO on TLR7. These results clearly demonstrate the sequence-dependent effects of 2′OMe AMOs, and indicate that the motif identified could be used to predict the inhibitory potential of 2′OMe AMOs. In this regard, enrichment analysis of the identified motif in AMOs targeting all human miRNAs in miRBaseV20 (in both 5′-3′ and 3′-5′ orientations) (41), identified 53 AMOs that are possibly highly inhibitory (Supplementary Table S3). Interestingly, miR-27a-3p and miR-301a-3p AMOs belong to this list of potential inhibitors. Although we did find miR-27a-3p to significantly inhibit TLR8 in human PBMCs, our experimental evidence does not support a strong inhibitory activity for miR-301a-3p in either mouse or human models. We speculate that this relates to the position of the motif at the 5′-end of the sequence, which precludes formation of secondary structures necessary for inhibitory activity; similar to what is seen with miR-122-5p short. Nonetheless, our predictions likely underrepresent the sum of inhibitory 2′OMe AMO sequences, as it only considers sequences with a close relationship to the motif (*P* < 0.001) and changing the stringency of prediction to *P* < 0.01 results in the selection of more than 500 AMOs. Furthermore, we anticipate that other inhibitory motifs exist. As such, none of the miRNAs present in Class 4, which were able to inhibit TLR7 in both 2′OMe and LNA/DNA chemistries, had the inhibitory motif, albeit most likely resulting from miRNA-independent activities. Indeed, while miR-19a-3p did not have the predicted highly inhibitory motif, it was able to repress TLR7 sensing independently of miR-19 targeting in miR-19-deficient cells (Figure 1). Moreover, microarray profiling of mRNA expression in BMMs after DOTAP-mediated LNA/DNA AMO transfection (for miR-19a-3p, miR-15a-5p and miR-34a-5p) did not result in a significant de-repression of genes targeted by these miRNAs according to specific hexamer enrichment analyses with DIANA-mirExtra (42) (data not shown). These results indicate that DOTAP-mediated transfection of AMOs inefficiently delivers them to the cytoplasm, while favouring endosomal delivery (20,43), thus pointing to miRNA-independent activities for Class 3 and 4 AMOs.

Although our predictions might be useful in identifying 2′OMe AMOs with greater TLR7 inhibitory activity, it is most likely that other parameters, including the secondary structure of the AMOs, also directly influence their activity. As shown in Figure 5, miR-224-5p mut2 AMO is more stable than the native miR-224-5p and miR-224-5p mut1 AMOs, and displayed the strongest TLR7 inhibitory activity. Given that 2′OMe RNAs have a strong affinity to TLR7 (13), it is reasonable to assume that the rules regulating their TLR7 activity are similar to that of immunostimulatory ssRNA, and that structure has an impact (20–21,44). We have previously shown that immunostimulatory motifs embedded in RNA with double-stranded regions were more potent activators of TLR8 (20,21). It can be speculated that the activity of 2′OMe inhibitory motifs is also partially dependent on surrounding sequences forming double-stranded structures. As such, a commercial 2′OMe AMO scaffold containing double-stranded 5′- and 3′-end loops might have increased inhibitory activity on TLR7/8 (36). Our analyses of truncated variants of miR-122-5p and miR-200a-3p 2′OMe AMOs also show that disruption of the context of the inhibitory motif can significantly reduce its activity (Figure 4). The miR-122-5p AMO (reported as ‘15mer 2′OMe 5′inZEN, 3′ZEN’) was previously found to have similar miR-122–5p inhibitory activity in reported assays, compared to its full-length counterpart (7). Although the use of short 2′OMe AMOs should be optimized to make sure they retain most of their inhibitory activity (7), this approach could therefore help to limit secondary structure and motifs with inhibitory activity on TLR7 sensing.

We also observed significant inhibition of immunostimulatory ssRNA sensing by select LNA/DNA phosphorothioate AMOs from Classes 3 and 4. Although miRNA-based mechanisms could be at play for LNA/DNA AMOs targeting abundant miRNAs (such as miR-191-5p, miR-16-5p, miR-29a-3p or miR-100-5p), such effects can be ruled out for other AMOs of Class 3 targeting poorly abundant miR-224-5p, miR-331-3p, miR-134-5p or miR-31-5p. Such miRNA-independent TLR7 inhibition by these LNA/DNA phosphorothioate AMOs is rather in line with previous studies that reported the specific inhibition of R848 and immunostimulatory ssRNA-driven TLR7 activation, by phosphorothioate DNA oligonucleotide (ODN) in human HEK293 TLR7 cells and in mouse splenocytes (29,30). The TLR7-inhibitory activity of ODNs was much more potent with a phosphorothioate backbone than with a phosphodiesterase one (30), and was sequence dependent (29). Critically, a specific TLR7 inhibitory ODN (IRS661) substantially reduced immune responses to influenza virus, dengue-2 virus and bacterial tRNA (17,29,45). In our work, we confirm the sequence-dependent effect of LNA/DNA phosphorothioate AMOs on TLR7 in both human PBMCs (through modulation of IFN-α levels) and mouse BMMs. However, unlike for 2′OMe AMOs, we were not able to identify enriched motifs controlling the sequence-dependent inhibitory activity of LNA/DNA AMOs on TLR7. There was little overlap between the inhibition of TLR7 in mouse and that of TLR8 in human PBMCs, indicating different modes of action of LNA/DNA AMOs for each receptor. Our data also suggest a direct contribution for the phosphorothioate backbone in the sequence-dependent inhibition of TLR8 (Supplementary Figure S2A).

Collectively, our findings underline a potential for misinterpretation of results from experiments using AMOs aimed at defining the role of specific miRNAs in responses involving the innate immune system. This is of particular concern when using *in vivo* injection of naked or liposome-encapsulated AMOs, which encounter phagocytes expressing TLR7/8. Noteworthy, we found that miR-200a-3p and NC1 2′OMe AMO variants conjugated with a 3′ cholesterol group (commonly used for *in vivo* administration and referred to as antagomiRs (4)) were also strong inhibitors of TLR7/8, with marginally less activity than native sequences (Supplementary Figure S2B). Importantly, 2′OMe modifications have been shown to inhibit other innate immune sensors and effectors, including MDA5 or IFN-induced proteins with tetratricopeptide repeats (46,47). It is therefore very likely that 2′OMe AMOs will also interfere with the normal function of these innate immune regulators, in addition to their inhibitory role on TLR7/8 described here. Furthermore, our results suggest that sequence-dependent TLR7 inhibition by AMOs is not restricted to 2′OMe AMOs, and can also be seen with LNA/DNA phosphorothioate AMOs. Given the therapeutic potential of these molecules, a thorough characterization of their inhibitory off-target effects on the immune system is required to ensure minimal unintended immune suppression to avoid sensitizing of patients to intercurrent viral and bacterial infections.

## SUPPLEMENTARY DATA

Supplementary Data are available at NAR Online.

SUPPLEMENTARY DATA

## References

[1] Helwak A., Kudla G., Dudnakova T., Tollervey D. (2013). Mapping the human miRNA interactome by CLASH reveals frequent noncanonical binding. Cell.

[2] Selbach M., Schwanhausser B., Thierfelder N., Fang Z., Khanin R., Rajewsky N. (2008). Widespread changes in protein synthesis induced by microRNAs. Nature.

[3] Eulalio A., Huntzinger E., Izaurralde E. (2008). Getting to the root of miRNA-mediated gene silencing. Cell.

[4] Lennox K.A., Behlke M.A. (2011). Chemical modification and design of anti-miRNA oligonucleotides. Gene Ther..

[5] Hutvagner G., Simard M.J., Mello C.C., Zamore P.D. (2004). Sequence-specific inhibition of small RNA function. PLoS Biol..

[6] Meister G., Landthaler M., Dorsett Y., Tuschl T. (2004). Sequence-specific inhibition of microRNA- and siRNA-induced RNA silencing. RNA.

[7] Lennox K.A., Owczarzy R., Thomas D.M., Walder J.A., Behlke M.A. (2013). Improved performance of anti-miRNA oligonucleotides using a novel non-nucleotide modifier. Mol. Ther. Nucleic Acids.

[8] Friderici K., Kaehler M., Rottman F. (1976). Kinetics of Novikoff cytoplasmic messenger RNA methylation. Biochemistry.

[9] Kariko K., Buckstein M., Ni H., Weissman D. (2005). Suppression of RNA recognition by Toll-like receptors: the impact of nucleoside modification and the evolutionary origin of RNA. Immunity.

[10] Robbins M., Judge A., Liang L., McClintock K., Yaworski E., MacLachlan I. (2007). 2′-O-methyl-modified RNAs act as TLR7 antagonists. Mol. Ther..

[11] Sioud M., Furset G., Cekaite L. (2007). Suppression of immunostimulatory siRNA-driven innate immune activation by 2′-modified RNAs. Biochem. Biophys. Res. Commun..

[12] Zamanian-Daryoush M., Marques J.T., Gantier M.P., Behlke M.A., John M., Rayman P., Finke J., Williams B.R. (2008). Determinants of cytokine induction by small interfering RNA in human peripheral blood mononuclear cells. J. Interferon Cytokine Res..

[13] Hamm S., Latz E., Hangel D., Muller T., Yu P., Golenbock D., Sparwasser T., Wagner H., Bauer S. (2010). Alternating 2′-O-ribose methylation is a universal approach for generating non-stimulatory siRNA by acting as TLR7 antagonist. Immunobiology.

[14] Sarvestani S.T., Williams B.R., Gantier M.P. (2012). Human Toll-like receptor 8 can be cool too: implications for foreign RNA sensing. J. Interferon Cytokine Res..

[15] Kane M., Case L.K., Wang C., Yurkovetskiy L., Dikiy S., Golovkina T.V. (2011). Innate immune sensing of retroviral infection via Toll-like receptor 7 occurs upon viral entry. Immunity.

[16] Wang J.P., Bowen G.N., Padden C., Cerny A., Finberg R.W., Newburger P.E., Kurt-Jones E.A. (2008). Toll-like receptor-mediated activation of neutrophils by influenza A virus. Blood.

[17] Jockel S., Nees G., Sommer R., Zhao Y., Cherkasov D., Hori H., Ehm G., Schnare M., Nain M., Kaufmann A. (2012). The 2′-O-methylation status of a single guanosine controls transfer RNA-mediated Toll-like receptor 7 activation or inhibition. J. Exp. Med..

[18] Gantier M.P., Irving A.T., Kaparakis-Liaskos M., Xu D., Evans V.A., Cameron P.U., Bourne J.A., Ferrero R.L., John M., Behlke M.A. (2010). Genetic modulation of TLR8 response following bacterial phagocytosis. Hum. Mutat..

[19] Heil F., Hemmi H., Hochrein H., Ampenberger F., Kirschning C., Akira S., Lipford G., Wagner H., Bauer S. (2004). Species-specific recognition of single-stranded RNA via toll-like receptor 7 and 8. Science.

[20] Gantier M.P., Tong S., Behlke M.A., Irving A.T., Lappas M., Nilsson U.W., Latz E., McMillan N.A., Williams B.R. (2010). Rational design of immunostimulatory siRNAs. Mol. Ther..

[21] Gantier M.P., Tong S., Behlke M.A., Xu D., Phipps S., Foster P.S., Williams B.R. (2008). TLR7 is involved in sequence-specific sensing of single-stranded RNAs in human macrophages. J. Immunol..

[22] Tluk S., Jurk M., Forsbach A., Weeratna R., Samulowitz U., Krieg A.M., Bauer S., Vollmer J. (2009). Sequences derived from self-RNA containing certain natural modifications act as suppressors of RNA-mediated inflammatory immune responses. Int. Immunol..

[23] Ventura A., Young A.G., Winslow M.M., Lintault L., Meissner A., Erkeland S.J., Newman J., Bronson R.T., Crowley D., Stone J.R. (2008). Targeted deletion reveals essential and overlapping functions of the miR-17 through 92 family of miRNA clusters. Cell.

[24] Clausen B.E., Burkhardt C., Reith W., Renkawitz R., Forster I. (1999). Conditional gene targeting in macrophages and granulocytes using LysMcre mice. Transgenic. Res..

[25] Gantier M.P., Williams B.R. (2010). Monitoring innate immune recruitment by siRNAs in mammalian cells. Methods Mol. Biol..

[26] Gantier M.P. (2013). Strategies for designing and validating immunostimulatory siRNAs. Methods Mol. Biol..

[27] Gantier M.P., Stunden H.J., McCoy C.E., Behlke M.A., Wang D., Kaparakis-Liaskos M., Sarvestani S.T., Yang Y.H., Xu D., Corr S.C. (2012). A miR-19 regulon that controls NF-kappaB signaling. Nucleic Acids Res..

[28] Gorden K.B., Gorski K.S., Gibson S.J., Kedl R.M., Kieper W.C., Qiu X., Tomai M.A., Alkan S.S., Vasilakos J.P. (2005). Synthetic TLR agonists reveal functional differences between human TLR7 and TLR8. J. Immunol..

[29] Barrat F.J., Meeker T., Gregorio J., Chan J.H., Uematsu S., Akira S., Chang B., Duramad O., Coffman R.L. (2005). Nucleic acids of mammalian origin can act as endogenous ligands for Toll-like receptors and may promote systemic lupus erythematosus. J. Exp. Med..

[30] Gorden K.K., Qiu X., Battiste J.J., Wightman P.P., Vasilakos J.P., Alkan S.S. (2006). Oligodeoxynucleotides differentially modulate activation of TLR7 and TLR8 by imidazoquinolines. J. Immunol..

[31] Zuker M. (2003). Mfold web server for nucleic acid folding and hybridization prediction. Nucleic Acids Res..

[32] Gantier M.P. (2013). The not-so-neutral role of microRNAs in neutrophil biology. J. Leukoc. Biol..

[33] Haussecker D. (2012). The business of RNAi therapeutics in 2012. Mol. Ther. Nucleic Acids.

[34] Janssen H.L., Reesink H.W., Lawitz E.J., Zeuzem S., Rodriguez-Torres M., Patel K., van der Meer A.J., Patick A.K., Chen A., Zhou Y. (2013). Treatment of HCV infection by targeting microRNA. N. Engl. J. Med..

[35] Hagedorn P.H., Yakimov V., Ottosen S., Kammler S., Nielsen N.F., Hog A.M., Hedtjarn M., Meldgaard M., Moller M.R., Orum H. (2013). Hepatotoxic potential of therapeutic oligonucleotides can be predicted from their sequence and modification pattern. Nucleic Acid Ther..

[36] Vermeulen A., Robertson B., Dalby A.B., Marshall W.S., Karpilow J., Leake D., Khvorova A., Baskerville S. (2007). Double-stranded regions are essential design components of potent inhibitors of RISC function. RNA.

[37] Lennox K.A., Behlke M.A. (2010). A direct comparison of anti-microRNA oligonucleotide potency. Pharm. Res..

[38] Judge A.D., Bola G., Lee A.C., MacLachlan I. (2006). Design of noninflammatory synthetic siRNA mediating potent gene silencing in vivo. Mol. Ther..

[39] Hornung V., Guenthner-Biller M., Bourquin C., Ablasser A., Schlee M., Uematsu S., Noronha A., Manoharan M., Akira S., de Fougerolles A. (2005). Sequence-specific potent induction of IFN-alpha by short interfering RNA in plasmacytoid dendritic cells through TLR7. Nat. Med..

[40] Bailey T.L., Elkan C. (1994). Fitting a mixture model by expectation maximization to discover motifs in biopolymers. Proc. Int. Conf. Intell. Syst. Mol. Biol..

[41] Grant C.E., Bailey T.L., Noble W.S. (2011). FIMO: scanning for occurrences of a given motif. Bioinformatics.

[42] Alexiou P., Maragkakis M., Papadopoulos G.L., Simmosis V.A., Zhang L., Hatzigeorgiou A.G. (2010). The DIANA-mirExTra web server: from gene expression data to microRNA function. PLoS One.

[43] Sioud M. (2005). Induction of inflammatory cytokines and interferon responses by double-stranded and single-stranded siRNAs is sequence-dependent and requires endosomal localization. J. Mol. Biol..

[44] Sarvestani S.T., Tate M.D., Moffat J.M., Jacobi A.M., Behlke M.A., Miller A.R., Beckham S.A., McCoy C.E., Chen W., Mintern J.D. (2014). Inosine-mediated modulation of RNA sensing by Toll-like receptor 7 (TLR7) and TLR8. J. Virol..

[45] Wang J.P., Liu P., Latz E., Golenbock D.T., Finberg R.W., Libraty D.H. (2006). Flavivirus activation of plasmacytoid dendritic cells delineates key elements of TLR7 signaling beyond endosomal recognition. J. Immunol..

[46] Zust R., Cervantes-Barragan L., Habjan M., Maier R., Neuman B.W., Ziebuhr J., Szretter K.J., Baker S.C., Barchet W., Diamond M.S. (2011). Ribose 2′-O-methylation provides a molecular signature for the distinction of self and non-self mRNA dependent on the RNA sensor Mda5. Nat. Immunol..

[47] Daffis S., Szretter K.J., Schriewer J., Li J., Youn S., Errett J., Lin T.Y., Schneller S., Zust R., Dong H. (2010). 2′-O methylation of the viral mRNA cap evades host restriction by IFIT family members. Nature.

