# Supplementary material for: Sequence-dependent off-target inhibition of TLR7/8 sensing by synthetic microRNA inhibitors
Source: Nucleic Acids Res. 2014 Dec 24;43(2):1177–88. doi: 10.1093/nar/gku1343 (PMC4333393; doi:10.1093/nar/gku1343)
Supplement: SUPPLEMENTARY DATA [file supp_43_2_1177__index.html]

Sequence-dependent off-target inhibition of TLR7/8 sensing by synthetic microRNA inhibitors — SUPPLEMENTARY DATA 

# Sequence-dependent off-target inhibition of TLR7/8 sensing by synthetic microRNA inhibitors

## SUPPLEMENTARY DATA

**Files in this Data Supplement:**

- SUPPLEMENTARY DATA
