# Supplementary material for: Sequence-dependent off-target inhibition of TLR7/8 sensing by synthetic microRNA inhibitors
Source: Nucleic Acids Res. 2014 Dec 24;43(2):1177–88. doi: 10.1093/nar/gku1343 (PMC4333393; doi:10.1093/nar/gku1343)
Supplement: SUPPLEMENTARY DATA [file supp_gku1343_nar-02326-y-2014-File007.pdf]

Supplementary Data

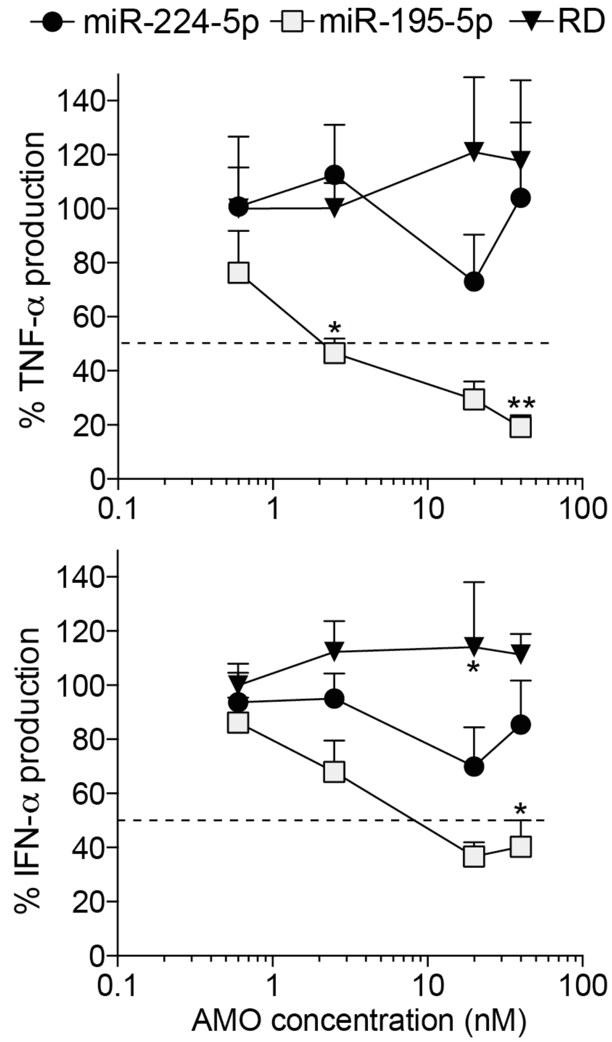

Supplementary Figure S1. Dose-dependent inhibition of TLR7/8 by 2'OMe AMOs. Human PBMCs were pre-treated for 45 min with the indicated dose of AMO or RD, and stimulated overnight with 180 nM of B-406AS-1. TNF-α and IFN-α levels were measured in supernatants by ELISA. The data are averaged from three independent experiments in different blood donors, in biological triplicate. Percentages of cytokine production compared to the 0.6 nM RD+B-406AS-1 condition are given. Ordinary one-way ANOVA with Dunnett's multiple comparison tests to the miR-224-5p+B-406AS-1 condition are shown. Unless otherwise indicated, differences were not significant. SEM is shown.

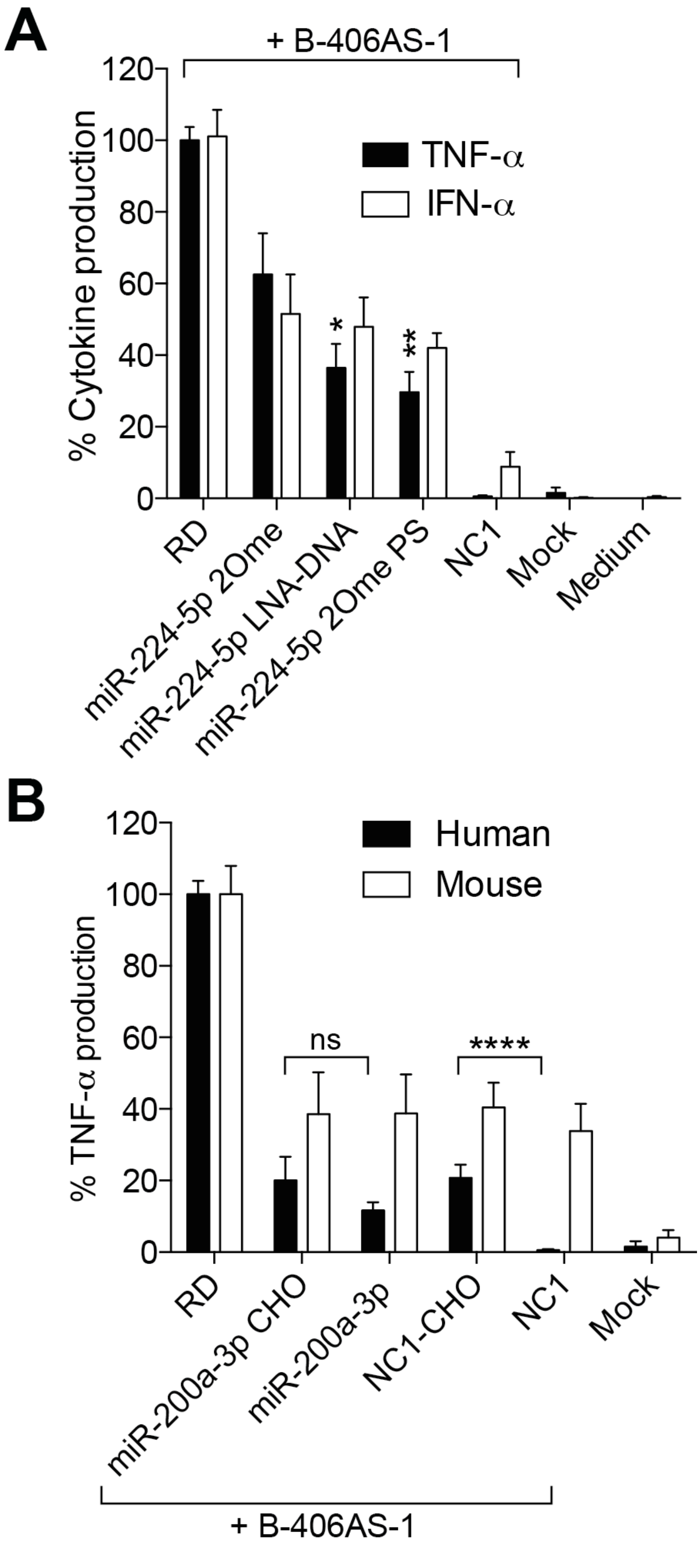

Supplementary Figure S2. Impact of chemistry on 2'OMe AMO inhibitory activity. Human PBMCs (**A, B**) and mouse primary BMMs (**B**) pre-treated with 40 nM of the indicated AMO for 45 min were stimulated overnight with 180 nM of B-406AS-1. Human/mouse TNF-α and IFN-α levels were measured in supernatants by ELISA. PS: phosphorothioate modified. CHO: 3'Cholesterol-modified. Percentages of cytokine production compared to the RD+B-406AS-1 condition are given. The data are averaged from a minimum of two independent experiments in three blood donors or three mice, in biological triplicate. Unpaired *t*-tests comparing miR-224-5p 2'OMe+B-406AS-1 (**A**) or miR-200a-3p/NC1+B-406AS-1 (**B**) and indicated conditions are shown ("ns": not significant).

Supplementary Table S1. AMO sequences used in this study

Human miRNA target	AMO sequence 2'OMe	AMO sequence LNA/DNA
miR-92a-3p	AzCAGGCCGGGACAAGUGCAAUz	NA
NC1	GzCGUAUUAUAGCCGAUUAACGzA	NA
miR-17-5p	CzUACCUGCACUGUAAGCACUUUz	c*+T*a*c*+C*t*g*+C*a*c*+T*g*t*+A*a*g*+C*a*c*+T*t*t*+G
miR-19a-3p	UzCAGUUUUGCAUAGAUUUGCACz	t*c*+A*g*t*+T*t*t*+G*c*a*+T*a*g*+A*t*t*+T*g*c*+A*c*a
miR-18a-5p	CzUAUCUGCACUAGAUGCACCUUz	c*+T*a*t*+C*t*g*+C*a*c*+T*a*g*+A*t*g*+C*a*c*+C*t*t*+A
miR-224-5p	AzACGGAACCACUAGUGACUUz	a*a*+C*g*g*+A*a*c*+C*a*c*+T*a*g*+T*g*a*+C*t*t*+G
miR-210-3p	UzCAGCCGCUGUCACACGCACAz	t*c*+A*g*c*+C*g*c*+T*g*t*+C*a*c*+A*c*g*+C*a*c*+A*g
miR-200c-3p	UzCCAUCAUUACCCGGCAGUAUUz	t*+C*c*a*+T*c*a*+T*t*a*+C*c*c*+G*g*c*+A*g*t*+A*t*t*+A
miR-301a-3p	GzCUUUGACAAUACUAUUGCACUz	g*c*+T*t*t*+G*a*c*+A*a*t*+A*c*t*+A*t*t*+G*c*a*+C*t*g
miR-331-3p	UzUCUAGGAUAGGCCCAGGGGz	t*+T*c*t*+A*g*g*+A*t*a*+G*g*c*+C*c*a*+G*g*g*+G*c
miR-125b-5p	UzCACAAGUUAGGGUCUCAGGGz	t*+C*a*c*+A*a*g*+T*t*a*+G*g*g*+T*c*t*+C*a*g*+G*g*a
miR-146a-5p	AzACCCAUGGAAUUCAGUUCUCz	a*a*+C*c*c*+A*t*g*+G*a*a*+T*t*c*+A*g*t*+T*c*t*+C*a
miR-126-3p	CzGCAUUAUUACUCACGGUACGz	c*g*+C*a*t*+T*a*t*+T*a*c*+T*c*a*+C*g*g*+T*a*c*+G*a
miR-134-5p	CzCCCUCUGGUCAACCAGUCACz	c*c*+C*c*t*+C*t*g*+G*t*c*+A*a*c*+C*a*g*+T*c*a*+C*a
miR-200a-3p	AzCAUCGUUACCAGACAGUGUUz	a*+C*a*t*+C*g*t*+T*a*c*+C*a*g*+A*c*a*+G*t*g*+T*t*a
miR-191-5p	CzAGCUGCUUUUGGGAUUCCGUUz	c*+A*g*c*+T*g*c*+T*t*t*+T*g*g*+G*a*t*+T*c*c*+G*t*t*+G
miR-200b-3p	UzCAUCAUUACCAGGCAGUAUUz	t*c*+A*t*c*+A*t*t*+A*c*c*+A*g*g*+C*a*g*+T*a*t*+T*a
miR-31-5p	AzGCUAUGCCAGCAUCUUGCCz	a*g*+C*t*a*+T*g*c*+C*a*g*+C*a*t*+C*t*t*+G*c*c*+T
miR-122-5p	CzAAACACCAUUGUCACACUCCz	c*+A*a*a*+C*a*c*+C*a*t*+T*g*t*+C*a*c*+A*c*t*+C*c*a
miR-100-5p	CzACAAGUUCGGAUCUACGGGUz	c*+A*c*a*+A*g*t*+T*c*g*+G*a*t*+C*t*a*+C*g*g*+G*t*t
miR-155-5p	AzCCCCUAUCACGAUUAGCAUUAz	a*+C*c*c*+C*t*a*+T*c*a*+C*g*a*+T*t*a*+G*c*a*+T*t*a*a
miR-182-5p	AzGUGUGAGUUCUACCAUUGCCAAz	a*+G*t*g*+T*g*a*+G*t*t*+C*t*a*+C*c*a*+T*t*g*+C*c*a*+A*a
miR-195-5p	GzCCAAUAUUUCUGUGCUGCUz	g*+C*c*a*+A*t*a*+T*t*t*+C*t*g*+T*g*c*+T*g*c*+T*a
miR-15a-5p	CzACAAACCAUUAUGUGCUGCUz	c*+A*c*a*+A*a*c*+C*a*t*+T*a*t*+G*t*g*+C*t*g*+C*t*a
miR-24-3p	CzUGUUCCUGCUGAACUGAGCCz	c*+T*g*t*+T*c*c*+T*g*c*+T*g*a*+A*c*t*+G*a*g*+C*c*a
miR-16-5p	CzGCCAAUAUUUACGUGCUGCUz	c*g*+C*c*a*+A*t*a*+T*t*t*+A*c*g*+T*g*c*+T*g*c*+T*a
miR-34a-5p	AzCAACCAGCUAAGACACUGCCz	a*+C*a*a*+C*c*a*+G*c*t*+A*a*g*+A*c*a*+C*t*g*+C*c*a
miR-29a-3p	UzAACCGAUUUCAGAUGGUGCUz	t*+A*a*c*+C*g*a*+T*t*t*+C*a*g*+A*t*g*+G*t*g*+C*t*a
miR-27a-3p	GzCGGAACUUAGCCACUGUGAz	g*c*+G*g*a*+A*c*t*+T*a*g*+C*c*a*+C*t*g*+T*g*a*+A
miR-21-5p	UzCAACAUCAGUCUGAUAAGCUz	t*+C*a*a*+C*a*t*+C*a*g*+T*c*t*+G*a*t*+A*a*g*+C*t*a
miR-25-3p	UzCAGACCGAGACAAGUGCAAUz	t*c*+A*g*a*+C*c*g*+A*g*a*+C*a*a*+G*t*g*+C*a*a*+T*g
miR-224-5p mut1	AzACGGAACCAUUAGUGACUUz	NA
miR-224-5p mut2	AzACGGAACCAUUACUGACUUz	NA
miR-200a-3p mut1	AzCAUCGUUCCCAGACAGUGUUz	NA
miR-200a-3p mut2	AzCAUGGUUCCCCGACAGUGUUz	NA
miR-200c-3p mut	UzCCAUCAUUACCAGGCAGUAUUz	NA
miR-200a-3p short	UzUACCAGACAGUGUUz	NA
miR-122-5p short	CzCAUUGUCACACUCCz	NA
NC1 mut1	GzCGUCUUAUAGCCGAUUAACGzA	NA
NC1 mut2	GzCGUCUUCUAGCCGAUUAACGzA	NA
NC1 mut3	GzCGUCUUCUAGGCGAUUAACGzA	NA
miR-224-5p PS	AzA*C*G*G*A*A*C*C*A*C*U*A*G*U*G*A*C*U*Uz	NA
NC1-CHO	GzCGUAUUAUAGCCGAUUAACGzA/iSp18//3CholMEG/	NA
miR-200a-3p-CHO	AzCAUCGUUACCAGACAGUGUUz/iSp18//3CholMEG/	NA

AMOs were synthesised with the following modifications: lowercase for DNA, UPPERCASE for 2'OMe, +underlined for LNA, with the asterisks for phosphorothioate linkages. NA (not applicable) denotes AMOs used in Figure 1 – without LNA/DNA counterpart. "z" denotes ZEN groups (see *Materials and Methods*), "iSp18" refers to an internal spacer, while "3CholMEG" refers to a 3'Cholesterol group.

Supplementary Table S2

Name	Start	*p*-value	Site
miR-182-5pR	3	1.26e-06	ACCGUUACCA
miR-200a-3p	3	3.49e-06	AUCGUUACCA
miR-122-5pR	8	3.08e-05	ACUGUUACCA
miR-200b-3p	3	6.34e-05	AUCAUUACCA
miR-25-3p	5	1.07e-04	ACCGAGACAA
AMO-NC1R	8	2.03e-04	GCCGAUAUUA
miR-195-5pR	9	3.72e-04	GUCUUUAUAA

MEME analysis of all Class 2 inhibitory 2’OMe AMOs (3’-5’ orientation is defined by ‘R’), with enriched inhibitory motif and *P* value (39).

Supplementary Table S3. FIMO *in silico* prediction of AMOs with inhibitory motif based on human miRBaseV20 (40). AMO to the indicated miRNAs are in 5'-3' or 3'-5' (indicated with 'R') orientation.

Motif	Sequence name	Strand	Start	End	*P*-value	*q*-value	Matched sequence
1	hsa-miR-182-5pR	+	3	12	8.89E-07	0.0423	ACCGTTACCA
1	hsa-miR-200a-3p	+	3	12	1.96E-06	0.0423	ATCGTTACCA
1	hsa-miR-367-3pR	+	11	20	1.96E-06	0.0423	ATCGTTACCA
1	hsa-miR-183-5pR	+	3	12	1.23E-05	0.199	ACCGTGACCA
1	hsa-miR-122-5pR	+	8	17	1.92E-05	0.249	ACTGTTACCA
1	hsa-miR-141-3p	+	3	12	2.72E-05	0.263	ATCTTTACCA
1	hsa-miR-200b-3p	+	3	12	3.04E-05	0.263	ATCATTACCA
1	hsa-miR-4765R	+	12	21	3.25E-05	0.263	ATCGATACAA
1	hsa-miR-1306-3pR	+	5	14	4.14E-05	0.298	ACCGAGACCA
1	hsa-miR-2052	+	6	15	6.76E-05	0.438	ACTGTTATCA
1	hsa-miR-3923R	+	4	13	7.92E-05	0.467	ATCATTACAA
1	hsa-miR-200b-3pR	+	11	20	9.09E-05	0.49	ACCATTACTA
1	hsa-miR-25-3p	+	5	14	0.000104	0.509	ACCGAGACAA
1	hsa-miR-3591-5p	+	7	16	0.00011	0.509	GCCATTATCA
1	hsa-miR-4660R	+	3	12	0.000126	0.51	GTCGAGACCA
1	hsa-miR-4786-3pR	+	7	16	0.000126	0.51	GTCGAGACCA
1	hsa-miR-7975	+	5	14	0.000141	0.537	GCCGTGACTA
1	hsa-miR-24-1-5p	+	1	10	0.000155	0.556	ACTGATATCA
1	hsa-miR-4791R	+	1	10	0.000181	0.618	ACCTATACTA
1	hsa-miR-6506-5pR	+	14	23	0.000234	0.756	ACTTATACCA
1	hsa-miR-4762-3p	+	9	18	0.000274	0.824	ATCTTGATCA
1	hsa-miR-195-5pR	+	9	18	0.00028	0.824	GTCTTTATAA
1	hsa-miR-4325	+	3	12	0.000375	1	ACTGAGACAA
1	hsa-miR-101-5p	+	10	19	0.000396	1	ACTGTGATAA
1	hsa-miR-579-3p	+	8	17	0.000441	1	GTTTATACCA
1	hsa-miR-3913-5p	+	5	14	0.000469	1	ATCAAGATCA
1	hsa-miR-4779	+	3	12	0.000474	1	GCTTTTACTA
1	hsa-miR-3937R	+	12	21	0.000481	1	ATCGTTACCC
1	hsa-miR-6075R	+	11	20	0.000516	1	GCCGTAACCA
1	hsa-miR-548f-5p	+	5	14	0.000534	1	ACTGTGATTA
1	hsa-miR-4795-5pR	+	10	19	0.000543	1	ATTATTATAA
1	hsa-miR-1248	+	12	21	0.000553	1	GCTTATACAA
1	hsa-miR-1294R	+	9	18	0.000575	1	ACCGTAACAA
1	hsa-miR-132-3pR	+	11	20	0.000592	1	GTCGGTACCA
1	hsa-miR-92a-1-5p	+	10	19	0.000615	1	ACCGATCCCA
1	hsa-miR-184	+	1	10	0.000617	1	ACCCTTATCA
1	hsa-miR-5588-5pR	+	3	12	0.000621	1	ACCGTAATCA
1	hsa-miR-4518	+	8	17	0.000672	1	ACAGTTATCA
1	hsa-miR-3160-5p	+	6	15	0.000702	1	GCTGAGACTA
1	hsa-miR-8068	+	7	16	0.000702	1	ATCCTTACAA
1	hsa-miR-648R	+	10	19	0.000712	1	CCCGTGACCA
1	hsa-miR-548as-3p	+	10	19	0.000716	1	GTTAATACAA
1	hsa-miR-6883-5p	+	3	12	0.000723	1	ATCCATACCA
1	hsa-miR-331-5pR	+	2	11	0.000723	1	ATCCATACCA
1	hsa-miR-3668	+	1	10	0.000742	1	ATTTTGATCA
1	hsa-miR-4703-5pR	+	14	23	0.000756	1	GTTTATATCA
1	hsa-miR-301a-3p	+	1	10	0.000767	1	GCTTTGACAA
1	hsa-miR-301b	+	1	10	0.000767	1	GCTTTGACAA
1	hsa-miR-147aR	+	6	15	0.000767	1	ACCTTTACGA
1	hsa-miR-509-3-5p	+	12	21	0.000781	1	ACCGTTAGTA
1	hsa-miR-4657R	+	5	14	0.000868	1	ACCTTCACCA

1	hsa-miR-4775	+	5	14	0.000936	1	ACCGAAACAA
1	hsa-miR-182-5p	+	13	22	0.000949	1	ACCATTGCCA
1	hsa-miR-27a-3pR	+	8	17	0.000988	1	ACCGATTCAA
1	hsa-miR-27b-3pR	+	8	17	0.000988	1	ACCGATTCAA
